# Unveiling functionality and conducting two-sample mendelian randomization on WGCNA-identified oxidative stress-related hub genes in metabolic dysfunction-associated fatty liver disease

**DOI:** 10.1016/j.bbrep.2024.101829

**Published:** 2024-09-22

**Authors:** Qian Zhu, Jiaqi Liu, Wuxuan Mei, Changchun Zeng

**Affiliations:** a Department of Gastroenterology, Shenzhen Longhua District Central Hospital, Shenzhen, 518110, China; b Xianning Medical College, Hubei University of Science and Technology, Xianning, 437100, China; c Department of Medical Laboratory, Shenzhen Longhua District Central Hospital, Guangdong Medical University, Shenzhen, 518110, China

**Keywords:** MAFLD, Oxidative stress, Mendelian randomization, WGCNA, Gene co-expression network

## Abstract

Metabolic dysfunction-associated fatty liver disease (MAFLD) shows accelerated development under the impact of oxidative stress (OS). There is an imperative to identify OS-related biomarkers in MAFLD and explore their potential mechanistic insights. The objective of this study was to identify OS-related biomarkers in MAFLD and explore their potential mechanisms. DEG analysis was performed using GSE17470 and GSE24807 datasets. An investigative exploration utilizing WGCNA was executed to elucidate hub OS-related genes. The intersection of OS-related hub genes identified by WGCNA and DEGs was systematically employed for thorough analyses. A mendelian randomization (MR) study examined the causal effect of C-reactive protein (CRP) on MAFLD. 59 OS-related DEGs were identified in MAFLD. WGCNA revealed 100 OS-related hub genes in MAFLD. Sixteen OS-related genes have been delineated as critical components in MAFLD. Enrichment analyses, employing GO and KEGG pathways, revealed pathways enriched with these genes. Following PPI analyses, the highest-ranking ten hub genes demonstrating abnormal expression were determined. Ultimately, a two-sample MR analysis demonstrated a causal link between the hub gene CRP and the occurrence of MAFLD. In this study, we harnessed WGCNA to formulate a co-expression network and identified hub OS-related DEGs in MAFLD. Additionally, the hub gene CRP exhibited a significant correlation with the predisposition to MAFLD. These findings offer innovative perspectives on the applications of OS-associated genes in individuals afflicted with MAFLD.

## Highlights

• Discovered 59 DEGs linked to NAFLD-induced OS.
• WGCNA revealed 100 OS-related hub genes, offering novel perspectives.
• Comprehensive analyses (GO, KEGG, PPI) elucidated insights.
• MR study established CRP's causal link to NAFLD.
• Contributed to a novel understanding of OS in NAFLD.

## 1 Introduction

MAFLD presently attains recognition as the preeminent contributor to chronic hepatic disorders, displaying a widespread global incidence of 25 % [1]. MAFLD is the most prevalent chronic liver condition, with a high occurrence among obese children and adolescents, leading to an elevated risk of disease advancement. Maintaining a nutritious diet and engaging in physical exercise remain the foremost strategies for managing and preventing MAFLD in children and adolescents. Nutritional and dietary strategies primarily encompass low-carbohydrate, low-free-sugar, low-fructose, and low-lipid diets, along with promoting healthy eating habits and the potential use of eicosapentaenoic acid, docosahexaenoic acid, vitamins, cysteine, l-carnitine, cysteamine, and probiotics [2]. MAFLD includes a spectrum from simple steatosis (SS) to non-alcoholic steatohepatitis (NASH), featuring necroinflammation and a more accelerated fibrosis advancement than SS [3]. In contrast to SS, individuals afflicted with NASH face an elevated susceptibility to unfavorable hepatic consequences, encompassing cirrhosis, hepatic failure, and liver cancer [4]. Thus, it is paramount to grasp a thorough understanding of the mechanisms that underlie the initiation and progression of MAFLD.

Numerous studies have elucidated the association between oxidative stress (OS), stemming from the dysregulation of reactive oxygen species (ROS), and various pathological mechanisms, including MAFLD [5]. Cells can manage reactive species levels via protective strategies, such as excited state quenchers, antioxidant enzymes, electrophile conjugation, scavengers of free radicals, metal-ion chelation, as well as increased cytoprotective protein expression. Proper regulation of reactive species allows them to function as beneficial signaling molecules, fostering oxidative eustress, which contributes to physiological processes. Excessive reactive species can result in oxidative damage, contributing to conditions such as hemolytic anemia due to glucose-6-phosphate dehydrogenase deficiency, fructose-related MAFLD, and acetaminophen-induced liver toxicity [6]. In MAFLD, the consumption of an excessive amount of fat triggers an aberrant redox reaction, leading to a disruption in energy metabolism and the excessive generation of ROS. ROS in MAFLD inflict damage upon liver cells, generating non-parenchymal cells, including Kupffer cells (KCs) and hepatic stellate cells (HSCs), contributing to steatosis, fibrosis, and inflammation, propelling MAFLD towards NASH and HCC [7]. Furthermore, ROS facilitates lipid accumulation within hepatocytes and inflicts damage upon these hepatic cells. Fat accumulation results in elevated levels of lipopolysaccharides in the liver, triggering the polarization of KCs toward the M1 phenotype in MAFLD. Subsequently, these M1-polarized macrophages instigate the synthesis of inflammatory cytokines and ROS [8]. However, the complete explication of the OS dynamics within MAFLD remains elusive. Only a minute subset of OS-related genes has undergone rigorous investigation, establishing their indispensable contribution to the advancement of MAFLD. Thus, the fundamental goal of this study is to elucidate additional pivotal genes associated with OS, holding the potential to substantiate the fundamental mechanisms governing MAFLD.

Genomic advancements disclose a multitude of disease-associated genetic variants, highlighting the significance of Mendelian Randomization (MR) in evaluating causality via genetic data [9]. MR analysis substantially mitigates the impact of confounding factors, thereby yielding more dependable causal associations [10,11]. This is achieved through the utilization of single nucleotide polymorphisms (SNPs) as instrumental variables (IVs), thereby facilitating the evaluation of the causal interplay between various exposures and outcomes [12]. In this study, we employed MR analysis to examine the influence of the selected hub gene on MAFLD.

The current landscape reveals a deficiency in bioinformatic investigations, as the intricate mechanisms of OS genes in MAFLD remain unexplored. Hence, the primary objective of this study is to probe into the correlation between OS-related hub genes and MAFLD, contributing to the understanding of OS-related pathways as potential therapeutic targets. This not only enriches the current investigative landscape but also furnishes a valuable citation for considering OS-related genes as a potential avenue for therapeutic intervention in MAFLD. The paramount outcome of our study reveals the foremost ten pivotal hub genes, namely MYC, HIF1A, FOS, ESR1, CXCL12, SERPINE1, CCL5, CXCL10, CRP, and ERBB2. These markers hold promise for managing MAFLD effectively and deepening our understanding of the fundamental mechanisms linked to OS. Subsequently, an MR study was conducted to elucidate the causal association between the hub gene CRP and MAFLD. Our findings illuminate the potential involvement of the OS process in MAFLD pathology, introducing a revitalized perspective for the management of MAFLD.

## 2 Materials and methods

### 2.1 Data source

Multiple data sets are available for analysis, two gene expression profiles (GSE17470 and GSE24807), each encompassing a sample size greater than 10, were retrieved from the Gene Expression Omnibus (GEO) database (http://www.ncbi.nlm.nih.gov/geo/). OS-related genes were obtained from the gene set enrichment analysis (GSEA; https://www.gsea-msigdb.org/gsea/msigdb/index.jsp) [13] and GeneCards database (https://www.genecards.org/) [14].

### 2.2 Identification of DEGs

Initially, we employed R software (version R 4.3.1) to retrieve and preprocess data from the GSE17470 (N = 11) and GSE24807 (N = 17) datasets. Furthermore, DEG analysis was conducted utilizing the “limma" package. Subsequently, the " pheatmap" and “ggplot 2" R packages were applied to produce volcano plots and DEG expression heatmaps.

### 2.3 Weighted gene co-expression network analysis

WGCNA is frequently employed to delineate patterns of genetic associations across diverse samples, and it is valuable for pinpointing synergistic genomes. Considering the complex interplay between genomes and their impact on phenotypes, this approach proves adept at pinpointing potential markers. A gene co-expression network for MAFLD was established utilizing the “WGCNA" R package [15]. In deconstructing the intricate interplay among distinct modules and the pathophysiological mechanisms underlying MAFLD, we unequivocally identified the most pivotal module as the central gene, employing the robust methodology of WGCNA.

### 2.4 Functional and pathway enrichment analyses

To elucidate the biological functionalities and intrinsic mechanisms of genes, we employed the “clusterProfiler" R package to analyze the top 30 enrichment of Gene Ontology (GO) and Kyoto Encyclopedia of Genes and Genomes (KEGG) terms associated with the target genes. The threshold for both p-value and q-value was established at 0.05 [16].

### 2.5 Protein-protein interaction network analyses

The prognostication and visualization of molecular interaction and protein-protein interaction (PPI) networks were executed utilizing STRING 12.0 (https://cn.string-db.org/) and the Cytoscape software 3 platform. The Cytoscape software's degree algorithm was employed for the prioritization of pivotal top ten genes within the PPI networks.

### 2.6 Mendelian randomization

Summary-level data regarding CRP were sourced from a genome-wide association meta-analysis with the Genome-Wide Association Studies (GWAS) identifiers ukb-*d*-30710_raw and ebi-a-GCST90018730 [17]. Within the ukb-*d*-30710_raw dataset, a total of 13, 586, 004 SNPs were discerned. In the ebi-a-GCST90018730 dataset, 83,025 samples underwent analysis, revealing the identification of 12, 493, 987 SNPs. Primary outcome data were retrieved from a publicly accessible GWAS dataset with the identifiers ebi-a-GCST90091033 (https://gwas.mrcieu.ac.uk/datasets/ebi-a-GCST90091033/) and finn-b-NAFLD (https://gwas.mrcieu.ac.uk/datasets/finn-b-NAFLD/). The dataset ebi-a-GCST90091033 involved 778,614 individuals of European origin (8434 MAFLD and 770,180 healthy controls) and included 6,784,388 SNPs. Meanwhile, the dataset ebi-a-GCST90091033 featured 218,792 Europeans (894 MAFLD and 217,898 healthy controls) and identified 16, 380, 466 SNPs [18]. To circumvent linkage disequilibrium, all SNPs were subjected to clumping using a clump window (r2 = 0.001 and kb = 10,000), with a significance threshold set at p < 5 × 10–6. A two-sample MR approach was employed to investigate the potential causal relationship between a central gene and the risk of MAFLD, utilizing the definition of SNPs as instrumental variables (IVs). The hub gene data were extracted from publicly accessible Genome-Wide Association Study (GWAS) datasets. MR analysis was conducted using the “TwoSampleMR" package, with inverse variance weighting (IVW) employed to appraise the connection between the levels of the hub gene and the risk of MAFLD. For additional sensitivity analysis, MR-Egger was implemented [19].

## 3 Results

### 3.1 DEGs screening

**Fig. 1:**
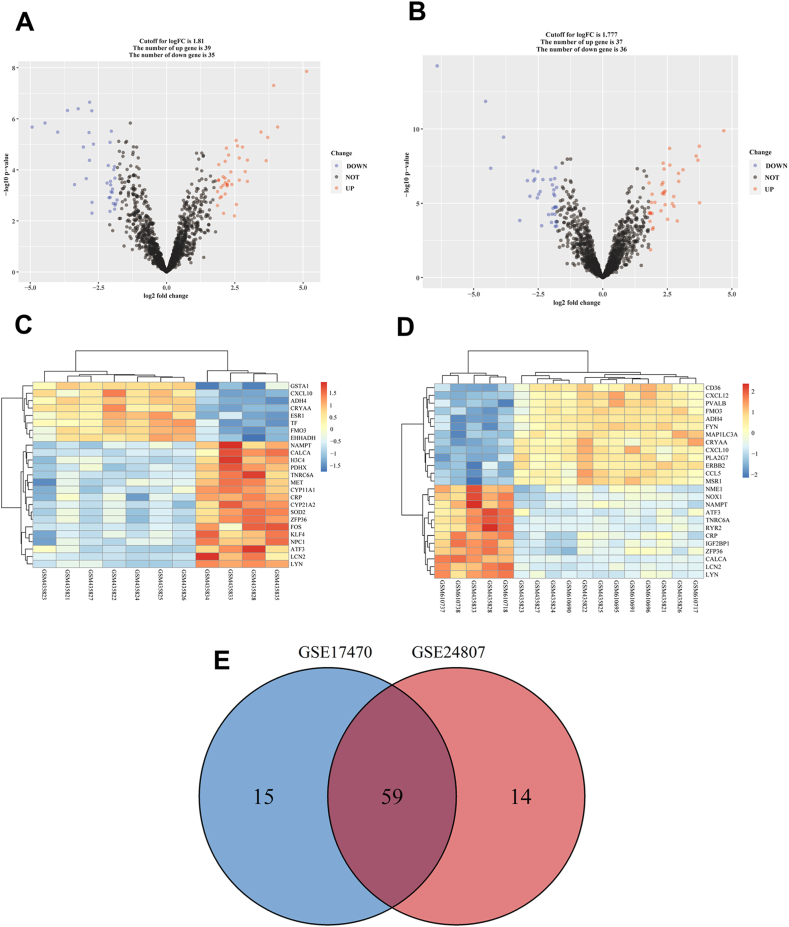
**DEGs analysis reveals OS-related distinct profiles between MAFLD and normal groups.** (A) Volcanic map illustrating OS-related DEGs in GSE17470. (B) Volcanic map illustrating OS-related DEGs in GSE24807. (C) Heat map illustrating OS-related DEGs of GSE17470. (D) Heat map illustrating OS-related DEGs of GSE24807. (E) The intersection of OS-related DEGs within the GSE17470 and GSE24807 datasets. OS: Oxidative stress; MAFLD: Metabolic dysfunction associated fatty liver disease; DEGs: Differentially expressed genes.

The MAFLD-related datasets (GSE17470 and GSE24807) were obtained from the GEO database. The GSE17470 (GPL2895) (N = 11) included 7 samples from NASH livers and 4 samples from normal healthy livers. The GSE24807 (GPL2895) (N = 17) comprised 12 NASH liver samples and 5 normal healthy liver samples. A collective assemblage of 1869 genes associated with OS was curated from the comprehensive datasets of GSEA and GeneCards (Supplementary Table 1). Specifically, 1509 OS-related genes were identified within the GeneCards database utilizing the keyword “oxidative stress" with a relevance score exceeding 6.0 and a gifts score surpassing 15.0. Additionally, 752 genes associated with OS were procured through the GSEA platform. Upon the preprocessing of the initial dataset, a total of 73 OS-related genes exhibiting differentially expressed patterns were identified from the GSE17470 dataset (Fig. 1A). Among these, 39 genes displayed an up-regulated pattern, while 35 genes exhibited down-regulation. In parallel, analysis of the GSE24807 dataset revealed 72 differential OS-related genes, consisting of 37 up-regulated and 36 down-regulated genes (Fig. 1B). In the GSE17470 dataset, a heat map was constructed to portray the DEGs linked with OS (Fig. 1C). A heat map was crafted to visually represent DEGs associated with OS within the GSE24807 dataset (Fig. 1D). The intersection of OS-related DEGs within the GSE17470 and GSE24807 datasets was enumerated as 59 (Supplementary Tables 2–4; Fig. 1E).

### 3.2 Elucidating MAFLD through the construction of a WGCNA network and identification of associated modules

**Fig. 2:**
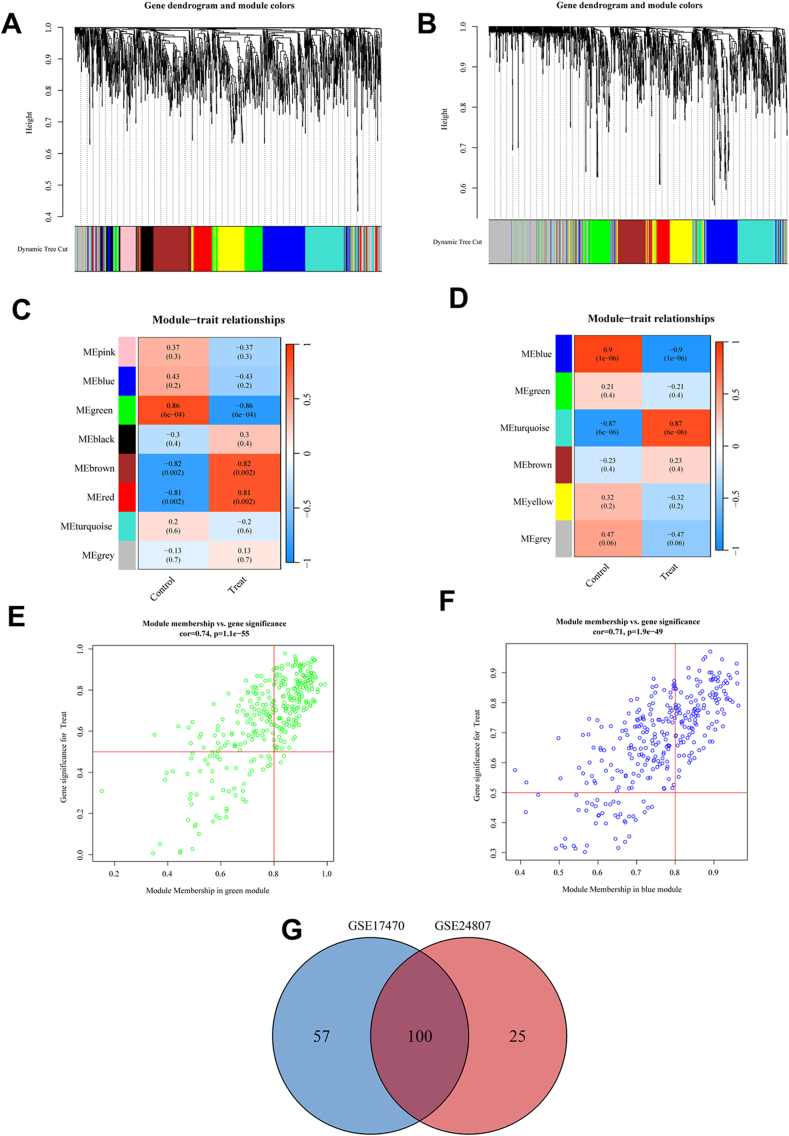
**MAFLD-associated gene modules identification in the GEO datasets through WGCNA.** (A) Dendrogram representing clustering of all genes in GSE17470 based on topological overlap matrix, with each branch corresponding to a gene and different colored branches denoting co-expression modules. (B) Dendrogram representing clustering of all genes in GSE24807 based on topological overlap matrix, with each branch corresponding to a gene and different colored branches denoting co-expression modules. (C)Module-trait heatmap displaying the correlation between clustering gene module and MAFLD in the GSE17470 dataset, including correlation coefficients and p Values for each module. (D) Module-trait heatmap indicating the correlation between clustering gene module and MAFLD in the GSE24807 dataset, with corresponding correlation coefficients and p Values for each module. (E) Scatter plot showing the strongest positive correlation of module green with MAFLD in the GSE17470 dataset. (F) Scatter plot demonstrating the strongest positive correlation of module blue with MAFLD in the GSE24807 dataset. (G) The overlapping set of OS-associated genes, as identified through WGCNA, across the GSE17470 and GSE24807 datasets. OS: Oxidative stress; MAFLD: Metabolic dysfunction associated fatty liver disease; WGCNA: Weighted gene co-expression network analysis. (For interpretation of the references to color in this figure legend, the reader is referred to the Web version of this article.)

To ascertain the potential association of gene modules with MAFLD, a meticulous WGCNA was conducted on the comprehensive set of OS-related genes derived from the GSE17470 (Supplementary Table 5) and GSE24807 (Supplementary Table 6) datasets intricately linked to MAFLD. Through the application of WGCNA for module categorization, we observed the presence of eight modules and six modules in the GSE17470 and GSE24807 datasets, respectively (Fig. 2A–D). In the GSE17470 datasets, the green module (r = 0.86, p = 6e−4), exhibiting the most noteworthy correlation with MAFLD, was identified as the key module (Fig. 2C). Simultaneously, a correlation analysis was undertaken between the green module membership and gene significance, revealing a substantial positive correlation (correlation coefficient = 0.74, p-value = 1.1e-55) (Fig. 2E). Furthermore, 157 hub genes were discerningly identified from the green module for subsequent analysis in the GSE17470 datasets. Given the correlation coefficient between modules and MAFLD, the blue module (r = 0.9, p = 1e−6) and turquoise module (r = 0.87, p = 6e-6), demonstrating the most substantial correlation with MAFLD, were recognized as the pivotal module in the GSE24807 datasets (Fig. 2D). In the GSE24807 datasets, correlation analyses between module membership and gene significance unveiled significant positive correlations for both the blue module (correlation coefficient = 0.71, p-value = 1.9e−49) and the turquoise module (correlation coefficient = 0.66, p-value = 1.2e−34) (Fig. 2F). Moreover, 125 hub genes were discerningly identified from the blue module for further analysis in the GSE24807 datasets. The intersection of OS-related genes as revealed by WGCNA within the datasets GSE17470 and GSE24807 was calculated to be 100 (Fig. 2G).

### 3.3 Functional enrichment analysis

**Fig. 3:**
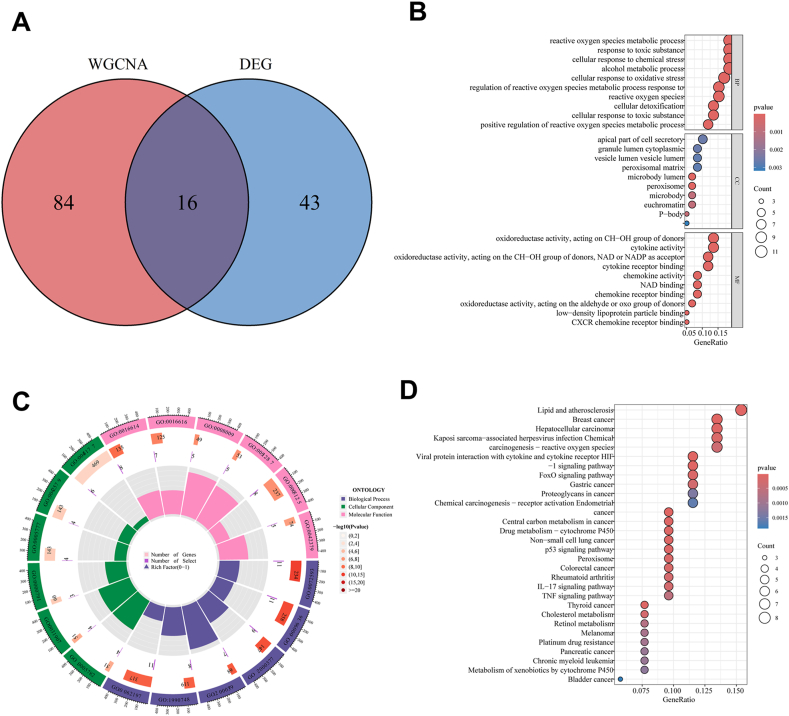
**Unveiling candidate hub OS-related genes in MAFLD through DEGs and WGCNA analysis.** (A) Venn diagram delineating the intersection of 16 candidate hub genes. (B) Bubble plot illustrating GO enrichment analysis of the candidate hub genes. (C) Circular plot showcasing GO enrichment analysis of the candidate hub genes. (D) Bubble plot presenting KEGG pathway analysis of the candidate hub genes. GO: Gene ontology; OS: Oxidative stress; MAFLD: Metabolic dysfunction associated fatty liver disease; WGCNA: Weighted gene co-expression network analysis. KEGG: Kyoto encyclopedia of genes and genomes.

The calculation of the overlap between OS-related DEGs and genes associated with OS derived from WGCNA was determined to be 16, including CYP21A2, SOD2, TNRC6A, ZFP36, CRP, NAMPT, MET, ADH1A, GADD45B, EPO, IGFBP1, RAB27A, VARS1, IGF2BP2, AIFM2, ODC1. These genes stand as prospective candidates warranting further investigation due to their potential roles in MAFLD (Fig. 3A). Subsequent research should investigate the feasibility of these genes as diagnostic tools and treatment targets by examining their expression patterns and biological roles. Furthermore, thorough clinical evaluations are required to verify their applicability in real-world scenarios and to determine how they might be incorporated into clinical routines. Pathway enrichment analyses were executed on the 16 candidate genes to gain a deeper insight into the fundamental mechanisms and functions of MAFLD. Significant involvement of these genes in signaling pathways, such as regulation of reactive oxygen species metabolic process, P-body, oxidoreductase activity, cytokine activity, and chemokine activity, was indicated by the results of GO enrichment analysis (Fig. 3B and C). The findings from KEGG analysis revealed the participation of genes in the IL-17 signaling pathway, HIF-1 signaling pathway, FoxO signaling pathway, p53 signaling pathway, and TNF signaling pathway (Fig. 3D).

### 3.4 PPI network analysis for hub genes

**Fig. 4:**
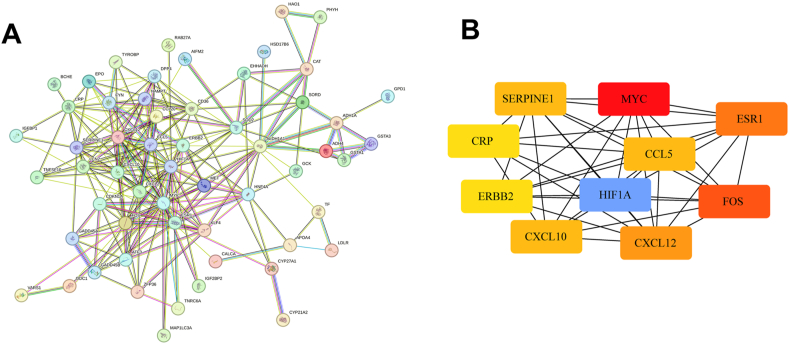
**Depicts the establishment of the PPI network.** (A) Depicts the PPI network featuring OS-related hub genes with significant overlap. (B) The extraction of pivotal genes within the interaction network was accomplished by employing the degree centrality algorithm. PPI: Protein-protein interaction; OS: Oxidative stress.

The STRING was employed to build a PPI network for the 16 overlapping candidate hub genes (Fig. 4A). Cytoscape software was then used to visually represent the top 10 abnormally expressed genes with the higher score. Concisely, MYC, HIF1A, FOS, ESR1, CXCL12, SERPINE1, CCL5, CXCL10, CRP, and ERBB2 were arranged based on the degree algorithm in MAFLD (Fig. 4B).

### 3.5 The causal relationship of CRP with the risk of MAFLD

**Fig. 5:**
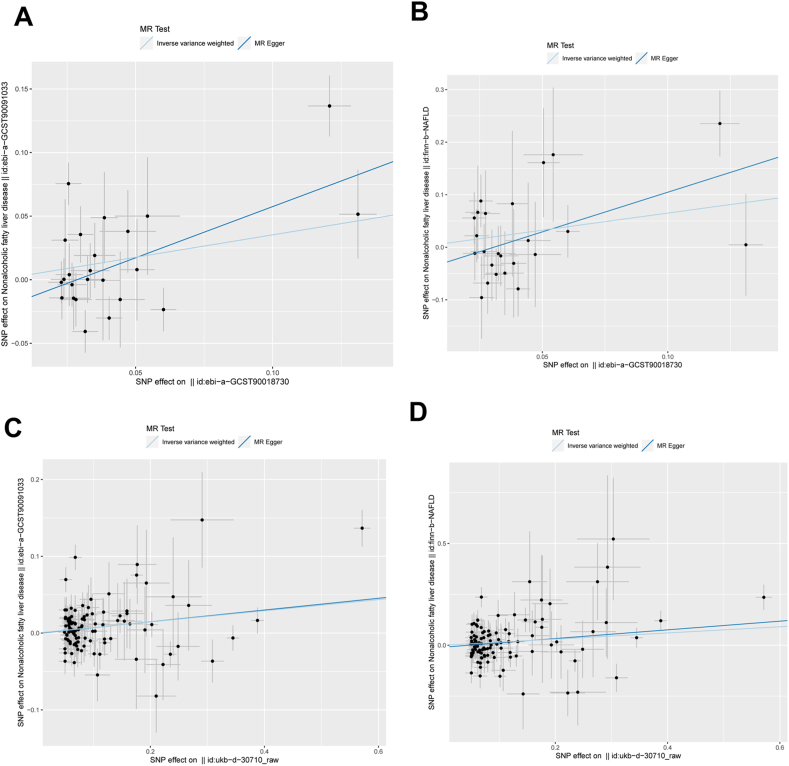
**The scatter plot distinctly portrays the significant causal influence of CRP on the risk of MAFLD in the MR study.** (A) The significant causal influence of CRP (ebi-a-GCST90018730) on the risk of MAFLD (ebi-a-GCST90091033); (B) The significant causal influence of CRP (ebi-a-GCST90018730) on the risk of MAFLD (finn-b-NAFLD); (C) The significant causal influence of CRP (ukb-*d*-30710_raw) on the risk of MAFLD (ebi-a-GCST90091033); (D) The significant causal influence of CRP (ukb-*d*-30710_raw) on the risk of MAFLD (finn-b-NAFLD). CRP: C-reactive protein; MAFLD: Metabolic dysfunction associated fatty liver disease; MR: mendelian randomization.

**Fig. 6:**
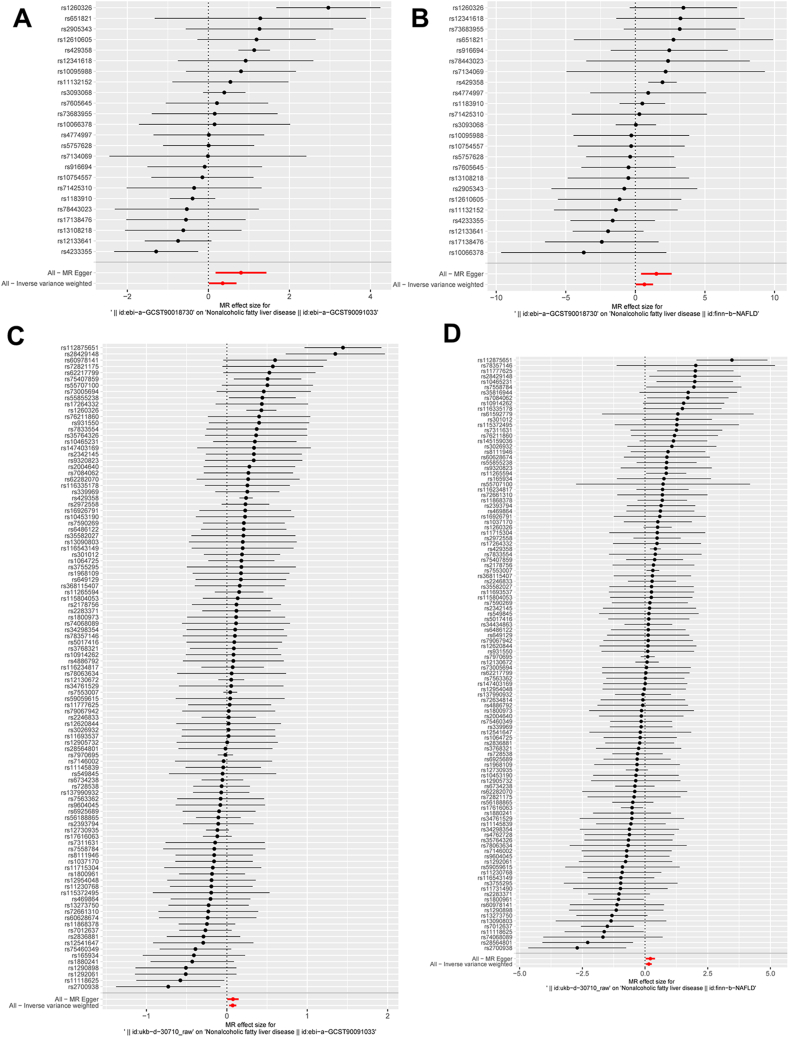
**The forest plot delineates the causal impact of each SNP on the susceptibility to MAFLD in the MR study.** (A) The causal impact of each SNP (ebi-a-GCST90018730) on the susceptibility to MAFLD (ebi-a-GCST90091033); (B) The causal impact of each SNP (ebi-a-GCST90018730) on the susceptibility to MAFLD (finn-b-NAFLD); (C) The causal impact of each SNP (ukb-*d*-30710_raw) on the susceptibility to MAFLD (ebi-a-GCST90091033); (D) The causal impact of each SNP (ukb-*d*-30710_raw) on the susceptibility to MAFLD (finn-b-NAFLD). MAFLD: Metabolic dysfunction associated fatty liver disease; SNPs: single-nucleotide polymorphisms; MR: mendelian randomization.

**Fig. 7:**
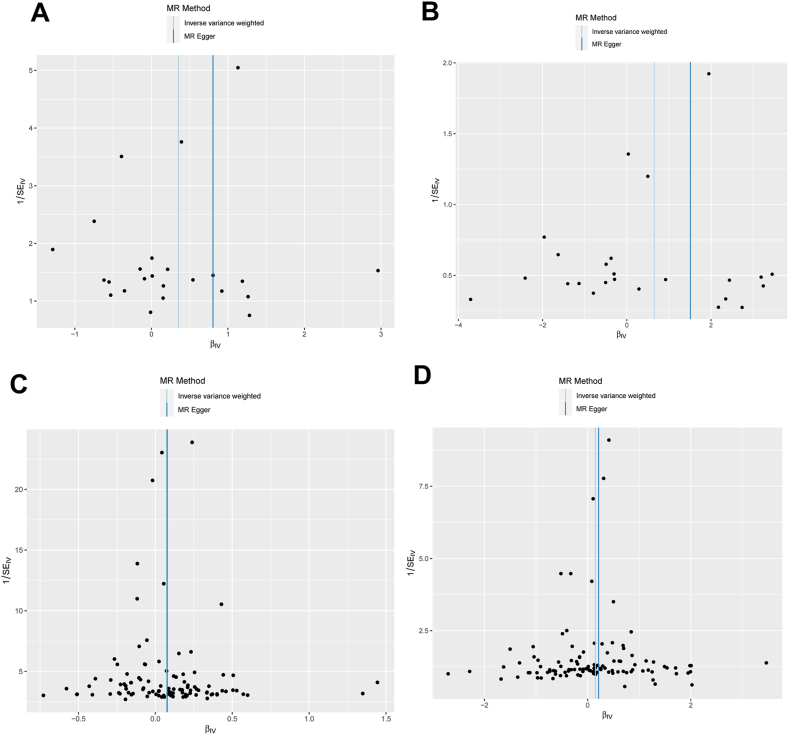
**Funnel plots were employed to visually represent the overarching heterogeneity of MR estimates regarding the impact of CRP on MAFLD.** (A) The overarching heterogeneity of MR estimates regarding the impact of CRP (ebi-a-GCST90018730) on MAFLD (ebi-a-GCST90091033); (B) The overarching heterogeneity of MR estimates regarding the impact of CRP (ebi-a-GCST90018730) on MAFLD (finn-b-NAFLD); (C) The overarching heterogeneity of MR estimates regarding the impact of CRP (ukb-*d*-30710_raw) on MAFLD (ebi-a-GCST90091033); (D) The overarching heterogeneity of MR estimates regarding the impact of CRP (ukb-*d*-30710_raw) on MAFLD (finn-b-NAFLD). CRP: C-reactive protein; MAFLD: Metabolic dysfunction associated fatty liver disease; MR: mendelian randomization.

**Fig. 8:**
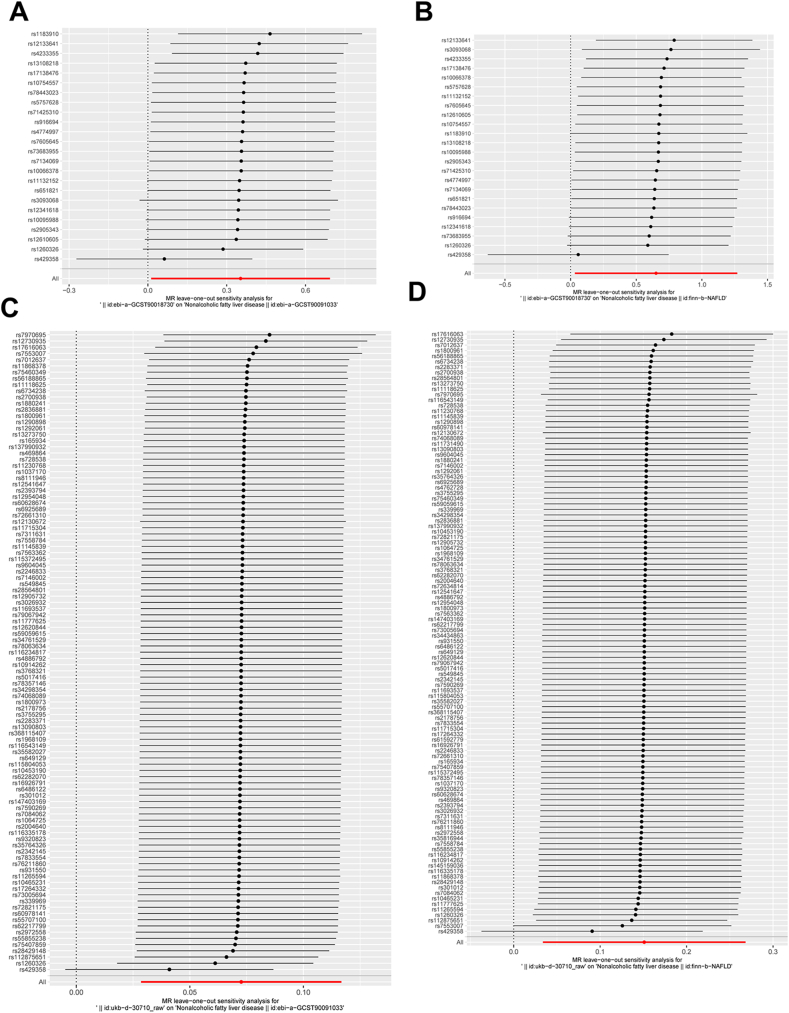
**Leave-one-out plot was applied to visualize the causal effect of CRP on the risk of MAFLD by leaving out one SNP in each iteration.** (A) The causal effect of CRP (ebi-a-GCST90018730) on the risk of MAFLD (ebi-a-GCST90091033) by leaving out one SNP in each iteration; (B) The causal effect of CRP (ebi-a-GCST90018730) on the risk of MAFLD (finn-b-NAFLD) by leaving out one SNP in each iteration; (C) The causal effect of CRP (ukb-*d*-30710_raw) on the risk of MAFLD (ebi-a-GCST90091033) by leaving out one SNP in each iteration; (D) The causal effect of CRP (ukb-*d*-30710_raw) on the risk of MAFLD (finn-b-NAFLD) by leaving out one SNP in each iteration. CRP: C-reactive protein; MAFLD: Metabolic dysfunction associated fatty liver disease; MR: mendelian randomization; SNPs: single-nucleotide polymorphisms.

In the two-way MR analysis, 261 SNPs were identified (Supplementary Table 7), employing CRP as the exposure (ebi-a-GCST90018730 and ukb-*d*-30710_raw) and MAFLD as the outcome (ebi-a-GCST90091033 and finn-b-NAFLD). No SNPs were identified as weak instrumental variables. Cochran's Q test did not detect substantial heterogeneity among the IVs, with a p-value exceeding 0.05. Utilizing the Inverse Variance Weighting (IVW) and Mendelian Randomization Egger (MR-Egger) methods, our analysis revealed a significant association between CRP and the risk of MAFLD (Fig. 5, Fig. 6; Table 1). The causal effect depicted by the funnel plot displayed an approximate symmetry (Fig. 7), and the MR Egger regression intercept did not reveal indications of horizontal pleiotropy. This additional evidence strengthens the argument that pleiotropy did not introduce bias to the observed causal effect (Table 2, Table 3). As illustrated in Fig. 8, a systematic reiteration of the MR analysis was conducted by iteratively excluding each SNP. Consistency in the results persisted, implying that the calculated outcomes of all SNPs consistently supported the significance of causality, validating the preceding MR results. In brief, our study primarily relies on IVW analysis and the MR-Egger method. The consistent findings indicate a positive causal association between CRP and MAFLD.

**Table 1:** Main results of MR analysis using MR Egger or IVW method.

Id.exposure	Id.outcome	Method	Nsnp	b	se	pval	lo_ci	up_ci	or	or_lci95	or_uci95
ebi-a-GCST90018730	ebi-a-GCST90091033	MR Egger	24	0.805	0.320	0.020	0.178	1.433	2.237	1.194	4.189
ebi-a-GCST90018730	ebi-a-GCST90091033	IVW	24	0.353	0.174	0.042	0.012	0.693	1.423	1.013	2.000
ebi-a-GCST90018730	finn-b-NAFLD	MR Egger	24	1.510	0.559	0.013	0.413	2.606	4.526	1.512	13.548
ebi-a-GCST90018730	finn-b-NAFLD	IVW	24	0.651	0.316	0.039	0.032	1.269	1.917	1.033	3.558
ukb-*d*-30710_raw	ebi-a-GCST90091033	MR Egger	103	0.077	0.036	0.035	0.006	0.147	1.080	1.006	1.158
ukb-*d*-30710_raw	ebi-a-GCST90091033	IVW	103	0.073	0.023	0.001	0.029	0.117	1.075	1.029	1.124
ukb-*d*-30710_raw	finn-b-NAFLD	MR Egger	110	0.213	0.093	0.024	0.030	0.396	1.238	1.031	1.486
ukb-*d*-30710_raw	finn-b-NAFLD	IVW	110	0.151	0.060	0.012	0.034	0.268	1.163	1.034	1.308

IVW: Inverse variance weighted.

**Table 2:** Heterogeneity analysis.

Id.exposure	Id.outcome	Method	Q	Q_df	Q_pval
ebi-a-GCST90018730	ebi-a-GCST90091033	MR Egger	57.813	22	4.68E-05
ebi-a-GCST90018730	ebi-a-GCST90091033	IVW	65.032	23	6.93E-06
ebi-a-GCST90018730	finn-b-NAFLD	MR Egger	23.487	22	3.75E-01
ebi-a-GCST90018730	finn-b-NAFLD	IVW	27.029	23	2.55E-01
ukb-*d*-30710_raw	ebi-a-GCST90091033	MR Egger	184.324	101	8.15E-07
ukb-*d*-30710_raw	ebi-a-GCST90091033	IVW	184.359	102	1.11E-06
ukb-*d*-30710_raw	finn-b-NAFLD	MR Egger	170.727	108	1.14E-04
ukb-*d*-30710_raw	finn-b-NAFLD	IVW	171.919	109	1.15E-04

IVW: Inverse variance weighted.

**Table 3:** Pleiotropy analysis.

Id.exposure	Id.outcome	Egger_intercept	se	pval
ebi-a-GCST90018730	ebi-a-GCST90091033	−0.023	0.014	0.112
ebi-a-GCST90018730	finn-b-NAFLD	−0.046	0.025	0.082
ukb-*d*-30710_raw	ebi-a-GCST90091033	−0.001	0.004	0.889
ukb-*d*-30710_raw	finn-b-NAFLD	−0.010	0.011	0.387

## 4 Discussion

MAFLD emerges as the primary instigator of hepatic lipidosis, epitomized by the dispersed deposition of lipids within the hepatic architecture [20,21]. MAFLD manifests as a continuum of liver pathologies, ranging from lipid accumulation to the intricate amalgamation of lipidosis and inflammation, and progressing to cirrhosis [22]. The nexus between MAFLD and metabolic syndrome is evident, embracing obesity, insulin resistance, type 2 diabetes, and hyperlipidemia [23,24]. It is of significance to observe that the global prevalence of obesity and its concomitant metabolic syndrome has propelled MAFLD into a prominent role as a causative factor for chronic liver diseases. The incidence of MAFLD is anticipated to persist in its upward trajectory, with MAFLD gradually emerging as the rapidly expanding catalyst for hepatocellular carcinoma [25]. Notwithstanding NASH's elevation to a significant global public health issue, the precise mechanistic underpinnings remain ambiguous. The urgency lies in the imperative exploration of MAFLD pathogenesis, the identification of associated biomarkers, the development of primary treatment strategies, and the formulation of preventive strategies.

The development of MAFLD is largely correlated with OS, lipid accumulation, lipotoxicity, and endoplasmic reticulum stress [26,27]. OS is indicative of the disproportion between the generation of ROS and the intricate efficiency of the antioxidant system in neutralization [28]. The initiation of OS by ROS, coupled with inflammation, emerges as a plausible and impactful mechanism conducive to tissue injury and hepatic cell death [29]. Mitochondrial aberrations, the exhaustion of glutathione (GSH), the attenuation of various antioxidant enzymes, the aggregation of leukocytes, the reduction in GSH-dependent antioxidant activity, and the onset of hepatic inflammation, are identified as the central contributors to the overproduction of ROS in MAFLD [7]. The extravagant generation of ROS dampens the effectiveness of antioxidative defense mechanisms in MAFLD, fostering an amplification of oxidative damage [30]. Given the intricate landscape of MAFLD, interwoven with a multitude of metabolic, genetic, and environmental elements, a thorough understanding of the distinct mechanistic contributions of OS to the pathogenesis of MAFLD is still elusive. In this paper, we discerned pivotal genes associated with OS in MAFLD and investigated the pathways associated with these crucial genes, contributing to a deeper understanding of the pivotal OS-related genes in the spectrum of MAFLD.

Recent investigations have surfaced employing bioinformatic tools to delve into the intricate mechanisms underpinning diseases within the MAFLD [30]. Among the routinely employed analytical methodologies, the DEG analysis stands out prominently [31]. Numerous studies have pinpointed pivotal genes in MAFLD, each delving into a specific facet and collectively advancing a more comprehensive understanding of MAFLD [[32], [33], [34], [35]]. In our investigation, the GEO database was employed to extract DEGs in MAFLD patients as opposed to normal individuals, to pinpoint robust biomarkers. The application of WGCNA featured prominently in the exploration of gene expression patterns, often employed concomitantly with the analysis of DEGs [[36], [37], [38]]. Within this investigation, an exhaustive exploration into the expression profile data was conducted via WGCNA. Subsequently, the identification of potential hub genes ensued through the convergence of DEGs and the outcomes of WGCNA. The pivotal genes identified in these investigations necessitate additional preclinical and clinical exploration. Thus, a discerning causative relationship between the hub gene CRP and MAFLD was innovatively elucidated through the meticulous application of MR analysis.

CRP, an acute-phase protein, emerges as a product of hepatic synthesis triggered by the release of inflammatory cytokines, notably tumor necrosis factor (TNF), interleukin 1 (IL-1), and interleukin 6 (IL-6) [39]. The monitoring of circulating CRP levels has been undertaken with a fluctuating level of efficacy in assessing the severity of diseases or forecasting their progression and eventual outcomes [40]. Heightened levels of CRP prove to be a valuable indicator for identifying patients' susceptibility to cardiovascular disease and specific cancers, offering guidance for therapeutic interventions [41,42]. Despite the liver being the primary source of CRP synthesis, attention should be given to the significant involvement of visceral fat, especially in adipose tissue, in CRP synthesis. Elevated levels of CRP are intricately tied to excessive body weight, attributed to the adipocyte-driven generation of TNF and IL-6, representing crucial elements in the stimulation of CRP [43]. The occurrence of MAFLD distinctly paralleled subclinical systemic inflammation, aligning with the observed elevation in CRP levels [44]. Elevated high-sensitivity CRP levels demonstrated a robust association with liver pathology, exhibiting commendable specificity in predicting fibrosis and steatosis in individuals with obesity [43]. In this paper, we established the connection between the hub gene CRP and MAFLD through the application of MR analysis.

The research faces some inherent limitations. The study heavily depends on publicly available bioinformatics data, which may not adequately reflect population diversity, possibly limiting the broader applicability of the findings. Even though the sample size is over 10, small sample sizes may result in biases or errors, pointing to the necessity for additional large-scale research to corroborate these results. A smaller sample size is acknowledged as a potential constraint in drawing broad conclusions. Moreover, a smaller sample size of the datasets could reduce the resolution of the gene co-expression network, restricting how broadly the conclusions can be applied. While the study offers some validation for the CRP gene through MR analysis, it lacks experimental evidence for hub genes, such as MYC, HIF1A, and FOS. Future research should incorporate techniques like qPCR, RNA sequencing, or proteomics to bolster the validation of our results. In addition, although pathways such as IL-17, HIF-1, p53, TNF, and FoxO are identified, this study does not extensively explain their mechanisms in the advancement of MAFLD. To advance mechanistic understanding, further research should probe the influence of specific genes on these pathways and their impact on the course of the disease. While the OS-related genes discovered in this research may hold clinical relevance, it remains speculative whether they can be used as diagnostic markers or therapeutic targets. To establish their clinical applicability, experimental research is required. Therefore, the findings should be considered exploratory, and additional validation through clinical studies is crucial.

## 5 Conclusion

In conclusion, our study utilized WGCNA to construct a co-expression network intricately associated with OS and delineate key DEGs linked to OS in MAFLD. The amalgamation of WGCNA with DEGs revealed 100 hub genes intricately associated with OS in MAFLD, with 16 identified as pivotal components in the OS nexus of MAFLD. Comprehensive analyses, encompassing GO, KEGG pathway enrichment, and PPI, provided insights into the functional roles and interconnected pathways enriched with these crucial genes. Furthermore, our MR study uncovered a causal relationship between the hub gene CRP and the occurrence of MAFLD, employing advanced genome-wide association study methodologies. Our innovative findings shed light on the potential applications of OS-associated genes in individuals afflicted with MAFLD.

## Data availability statement

The original contributions presented in the study are included in the article/Supplementary material, further inquiries can be directed to the corresponding authors.

## Funding

This present study was supported in part by the 10.13039/501100001809National Natural Science Foundation of China (grant number: 82,270,940).

## CRediT authorship contribution statement

**Qian Zhu:** Writing – original draft, Methodology, Conceptualization. **Jiaqi Liu:** Writing – original draft, Visualization, Software, Data curation. **Wuxuan Mei:** Writing – review & editing, Supervision, Investigation. **Changchun Zeng:** Writing – review & editing, Validation.

## Declaration of competing interest

The authors declare that they have no known competing financial interests or personal relationships that could have appeared to influence the work reported in this paper.
